# FDA-Approved Drugs with Potent In Vitro Antiviral Activity against Severe Acute Respiratory Syndrome Coronavirus 2

**DOI:** 10.3390/ph13120443

**Published:** 2020-12-04

**Authors:** Ahmed Mostafa, Ahmed Kandeil, Yaseen A. M. M. Elshaier, Omnia Kutkat, Yassmin Moatasim, Adel A. Rashad, Mahmoud Shehata, Mokhtar R. Gomaa, Noura Mahrous, Sara H. Mahmoud, Mohamed GabAllah, Hisham Abbas, Ahmed El Taweel, Ahmed E. Kayed, Mina Nabil Kamel, Mohamed El Sayes, Dina B. Mahmoud, Rabeh El-Shesheny, Ghazi Kayali, Mohamed A. Ali

**Affiliations:** 1 Center of Scientific Excellence for Influenza Viruses, National Research Centre, Giza 12622, Egypt; Ahmed.Kandeil@human-link.org (A.K.); Omnia.Abdelaziz@human-link.org (O.K.); Yasmin.Moatasim@human-link.org (Y.M.); Mahmoud.Shehata@human-link.org (M.S.); Mokhtar.Rizk@human-link.org (M.R.G.); noura.mahrous1995@gmail.com (N.M.); Sarah.Hussein@human-link.org (S.H.M.); gaballah09@gmail.com (M.G.); Ahmed.Nageh@human-link.org (A.E.T.); Ahmed.Elsayed@human-link.org (A.E.K.); minanabil56@yahoo.com (M.N.K.); mohameddiaaelsayes@outlook.com (M.E.S.); ra_eny@yahoo.com (R.E.-S.); 2 Organic & Medicinal Chemistry Department, Faculty of Pharmacy, University of Sadat City, Menoufia 32897, Egypt; yaseen.elshaier@fop.usc.edu.eg; 3 Department of Biochemistry & Molecular Biology, Drexel University College of Medicine, Philadelphia, PA 19102, USA; aaa396@drexel.edu; 4 Department of Microbiology and Immunology, Zagazig University, Zagazig 44519, Egypt; hishamabbas2008@gmail.com; 5 Pharmaceutics Department, National Organization for Drug Control and Research, Giza 12654, Egypt; dina_bahaa2007@yahoo.com; 6 Department of Epidemiology, Human Genetics, and Environmental Sciences, University of Texas, Houston, TX 77030, USA; 7 Human Link, Baabda 1109, Lebanon

**Keywords:** SARS-CoV-2, COVID-19, antiviral, virtual screening, drug repurposing

## Abstract

(1) Background: Drug repositioning is an unconventional drug discovery approach to explore new therapeutic benefits of existing drugs. Currently, it emerges as a rapid avenue to alleviate the COVID-19 pandemic disease. (2) Methods: Herein, we tested the antiviral activity of anti-microbial and anti-inflammatory Food and Drug Administration (FDA)-approved drugs, commonly prescribed to relieve respiratory symptoms, against Severe Acute Respiratory Syndrome Coronavirus 2 (SARS-CoV-2), the viral causative agent of the COVID-19 pandemic. (3) Results: Of these FDA-approved antimicrobial drugs, Azithromycin, Niclosamide, and Nitazoxanide showed a promising ability to hinder the replication of a SARS-CoV-2 isolate, with IC_50_ of 0.32, 0.16, and 1.29 µM, respectively. We provided evidence that several antihistamine and anti-inflammatory drugs could partially reduce SARS-CoV-2 replication in vitro. Furthermore, this study showed that Azithromycin can selectively impair SARS-CoV-2 replication, but not the Middle East Respiratory Syndrome Coronavirus (MERS-CoV). A virtual screening study illustrated that Azithromycin, Niclosamide, and Nitazoxanide bind to the main protease of SARS-CoV-2 (Protein data bank (PDB) ID: 6lu7) in binding mode similar to the reported co-crystalized ligand. Also, Niclosamide displayed hydrogen bond (HB) interaction with the key peptide moiety GLN: 493A of the spike glycoprotein active site. (4) Conclusions: The results suggest that Piroxicam should be prescribed in combination with Azithromycin for COVID-19 patients.

## 1. Introduction

Coronaviruses (CoVs) with positive-sense single-stranded RNA genome of non-segmented nature are divided according to phylogenetic clustering into four genera (alpha-, beta-, gamma-, and delta-CoVs) within subfamily Coronavirinae and family Coronaviridae of the Nidovirales order. The Beta-CoVs, of the greatest clinical importance to humans, are further subclassified into four lineages [1].

For many years, two alpha-CoVs (HCoV-229E, HCoV-NL63) and two beta-CoVs (HCoV-OC43 and HCoV-HKU1) were known to be associated with a mild and self-limiting respiratory infection in humans, namely the common cold. This list of low pathogenic coronaviruses was recently expanded by the addition of two highly pathogenic human beta-CoVs, the Severe Acute Respiratory Syndrome Coronavirus (SARS-CoV) in 2003 and the Middle East Respiratory Syndrome Coronavirus (MERS-CoV) in 2012 [2,3,4].

On 31 December 2019, a cluster of human infections in Wuhan city, Hubei province, China, of unknown etiology was reported to the World Health Organization (WHO) China Country Office. The infections were associated with elevated temperature, cough, shortness of breath, and pneumonia [5]. On 7 January 2020, the Chinese authorities attributed these respiratory infections to a new type of coronavirus. The new virus was designated firstly as 2019 novel coronavirus (2019-nCoV) and then Severe Acute Respiratory Syndrome Coronavirus 2 (SARS-CoV-2), but the disease itself is known as coronavirus disease 2019 (COVID-19). A few weeks later, the COVID-19 was vastly reported in all provinces in China and later expanded to all continents. As of 8 September 2020, COVID-19 is responsible for >27 million confirmed human cases in >215 countries and territories, with approximately 900,000 human deaths, but the actual number of cases is much higher due to asymptomatic infections [6,7]. The Phylogenic analysis of the ancestor of SARS-CoV-2 showed that this novel virus is more similar to the 2003 SARS-CoV and that both belong to lineage B of Beta-CoVs [8].

Despite that a great effort is currently running in the direction of vaccine development and drug repurposing, most of the clinically used drugs to relieve COVID-19 symptoms were based on clinical observations rather than experimental validations. To rapidly define potential therapeutic options against SARS-CoV-2, testing existing Food and Drug Administration (FDA)-licensed drugs for efficacy against novel viral pathogens represents a practical approach for anti-CoV screening. This could expedite the recommendation and/or implementation of those FDA-approved drugs with effective anti-COVID-19 activity in the treatment protocol [9,10].

Concerns have been raised that steroidal and non-steroidal anti-inflammatory drugs (SAIDs and NSAIDs, respectively) may be associated with an increased risk of adverse events when used in patients with acute viral respiratory infections, including COVID-19 [11,12], however, no clear evidence of severe adverse effects in patients with COVID-19 were reported [13]. On the other hand, antibiotics are commonly prescribed for treating respiratory bacterial infections. Besides, they are prescribed in viral infections based on clinical antiviral observations or to combat potential secondary bacterial infection [14]. The improper use of antibiotics to combat the COVID-19 pandemic will strengthen bacterial resistance and ultimately lead to more deaths during the crisis and beyond [15]. In this study, we investigated the impact of commonly prescribed anti-asthmatics, antibiotics, SAIDs, and NSAIDs, on the replication efficiency of SARS-CoV-2 virus in vitro in cell culture.

## 2. Results

### 2.1. Antiviral Activity Screening for Commonly Prescribed FDA-Approved Analgesics, Antipyretics, Anti-Inflammatory Drugs, and Antibiotics

The selected FDA-approved drugs were chosen (Table 1) based on different criteria, including their common prescription in Influenza-like illness (ILI) and in quarantine (clinic and self-isolation programs), and their application in treatment protocols for COVID-19 due to an observed improvement in illness-associated symptoms. The majority of the selected FDA-approved drugs are over the counter (OTC) medicines in developing countries, meaning that they can be bought without a prescription. This eases their intensive consumption by the public to treat mild to moderate ILI infections. Little is known about the antiviral activity of these predefined libraries of FDA-approved drugs.

### 2.2. Cytotoxicity and Antiviral Activity of Selected FDA-Approved Drugs

**Figure 1:**
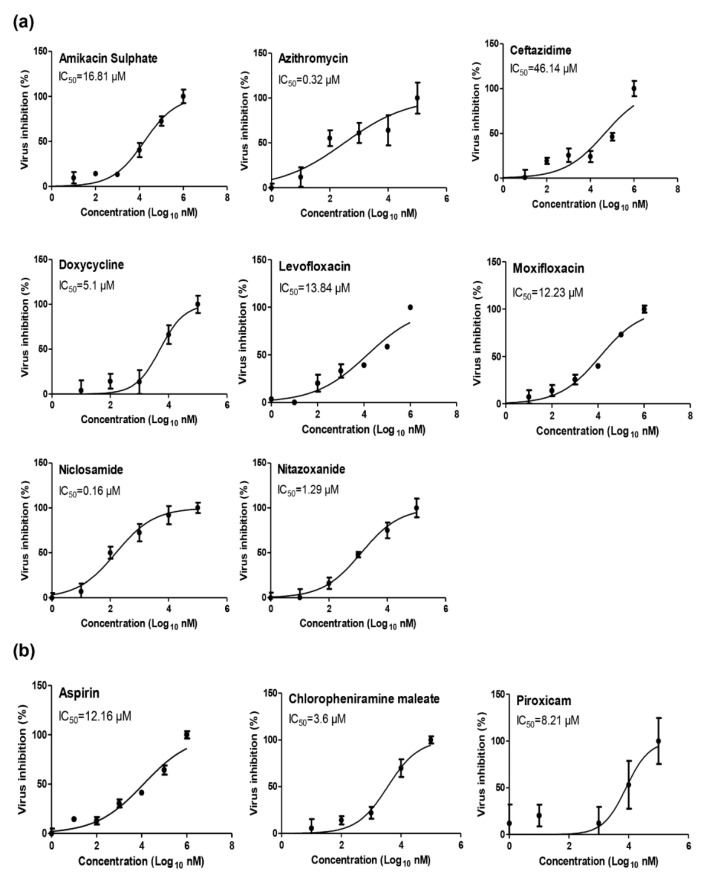
Dose-inhibition curves for anti-microbial and anti-inflammatory FDA-approved drugs with high selectivity indices against NRC-03-nhCoV. (**a**) Amikacin sulphate, Azithromycin, Ceftazidime, Doxycycline, Levofloxacin, Moxifloxacin, Niclosamide, and Nitazoxanide, (**b**) Aspirin, Chlorpheniramine maleate, and Piroxicam. Inhibitory concentration 50% (IC_50_) values were calculated using nonlinear regression analysis of GraphPad Prism software (version 5.01) by plotting log inhibitor versus normalized response (variable slope).

To identify the proper concentrations to define the antiviral activity of the selected drugs, half maximal cytotoxic concentration “CC_50_” was calculated by MTT assay for each individual drug (Table 1, Supplementary Figures S1 and S2). The antiviral screening revealed that a large number of the tested FDA-approved drugs exhibited a promising in vitro activity against NRC-03-nhCoV and have promising antiviral activities with a high selectivity index (≥100) for antiviral activity relative to cellular toxicity (Table 1, Figure 1, Supplementary Figures S3 and S4).

### 2.3. Mechanism of Anti-SARS-CoV-2 Activity for Promising FDA-Approved Drugs

The percent inhibition of various mechanisms of action are shown in Table 2. Interestingly, Azithromycin, Niclosamide, and Nitazoxanide have the least dual combinations of viral inhibitory effects on SARS-CoV-2 at different viral stages. Azithromycin has up to 51% virucidal effect, indicating that it acts directly on the virion and inactivates it. Additionally, it showed up to 31% inhibition at 1.3 µM concentration during the viral adsorption stage and negligible effect on viral replication. Niclosamide exhibited moderate virucidal effect with 37% viral inhibitory effect as well as 70% inhibitory effect on virus replication. A negligible reduction in viral inhibition was detected during application of viral adsorption mechanism. Nitazoxanide showed multiple inhibitory effects at the three stages but potency of its activity is mainly virucidal effect.

### 2.4. Molecular Modeling and Virtual Screening Study

#### 2.4.1. Molecular Docking Study

**Figure 2:**
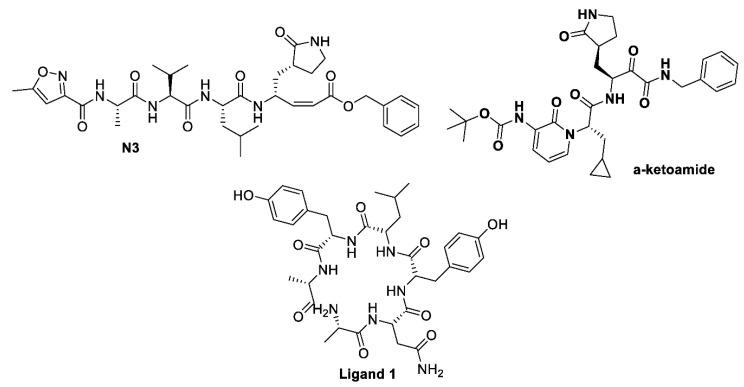
Chemical structure of ligands α-ketomaide and N3 for SARS-CoV-2 M^pro^ (PDB ID: 6lu7, 6y2f), and Ligand 1 for SARS-CoV-2 spike glycoprotein (PDB ID: 6vsb).

The X-ray crystal structure coordinates of SAR-CoV-2 main protease (M^pro^) were retrieved from protein data bank (PDB) ID: 6yef [16] and 6lu7 [17], in addition to the retrieved receptor for S glycoprotein (PDB ID:6vsb [18]), with their co-crystallized bounded ligands α-ketoamide, N3, and ligand 1, respectively (Figure 2). The docking study was performed using the OpenEye software (EON 2.3.3.4: OpenEye Scientific Software, Santa Fe, NM, USA. http://www.eyesopen.com). For the validation of the docking study, the co-crystal-bound ligands were redocked. Both structures exhibited high similarity and overlaid each other, as reported previously [16].

##### Docking with M^pro^ (PDB ID: 6lu7) of SARS-CoV-2

**Figure 3:**
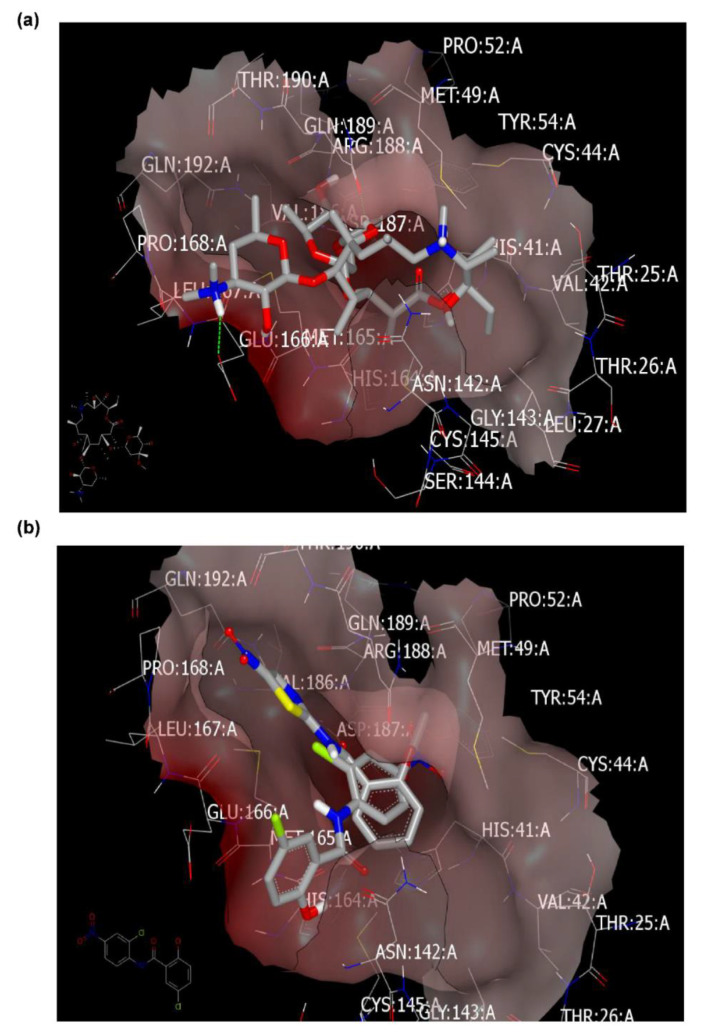
Visual representation by volumetric image display and analysis (VIDA) of docking with M^pro^ (PDB ID: 6lu7). (**a**) Azithromycin docked with the formation of two HBs (green color). (**b**) Niclosamide (grey color) and Nitazoxanide (thiazole ring with yellow-blue color) occupied the active site without detection of HB.

From the analysis of binding modes of Azithromycin, Niclosamide, and Nitazoxanide, as illustrated in Table 1, these drugs showed a correlation between their activity and their interaction with M^pro^ (PDB ID: 6lu7). Azithromycin (basic drug) fully occupied the receptor domains with the formation of two hydrogen bonds (HBs) with GLU:166A and GLN:189A (Figure 3a). Both amino acids interacted with the co-crystalized ligand as reported. Both Niclosamide and Nitazoxanide occupied the active site without detection of HB (Figure 3b). However, the salicyloyl moiety of Nitazoxanide overlays with the P-nitroaniline moiety of Niclosamide towards the receptor domain with ASP:187A-GLN:189A peptide part. The other groups of both drugs adopted different poses: the phenolic part of Niclosamide oriented closely toward the GLU:166A, MET:165A, and HIS:164A peptide cleft, especially in chlorine atoms and hydroxyl functionality. On the other hand, the thiazole ring adopted a position close to GLU:192A, LEU:176A, and VAL:186A cleft. This binding mode and pattern could explain the high activity of Niclosamide because of its capability to interact with the receptor via the most important key amino acids, especially GLU:166A and GLN:189A.

##### Docking with Spike Glycoprotein (PDB ID: 6vsb)

**Figure 4:**
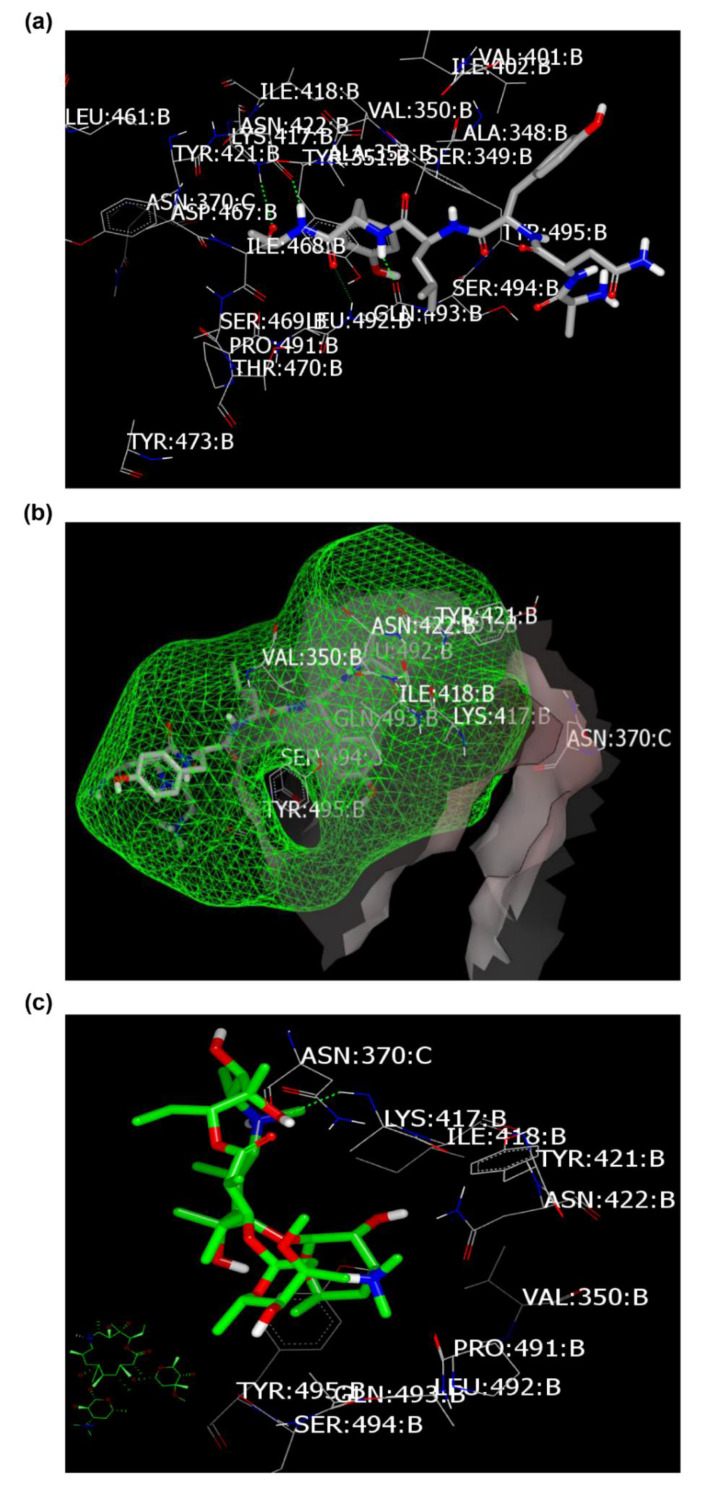

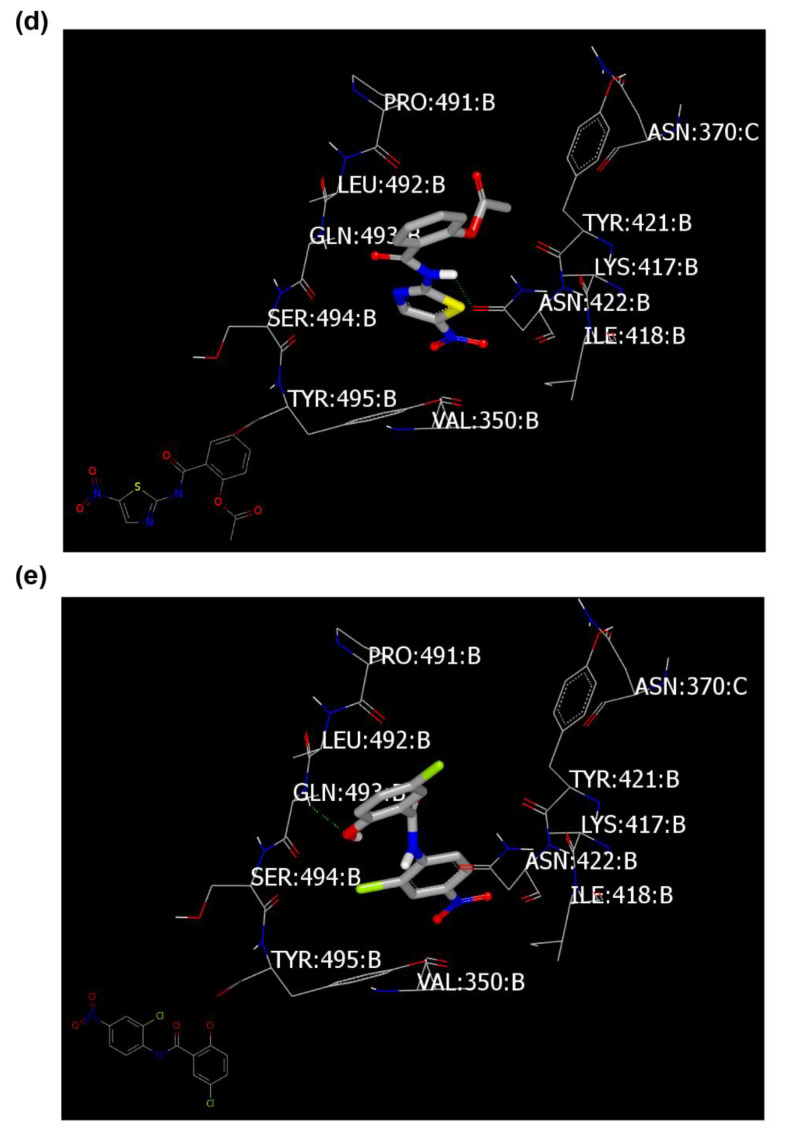
Visual representation by VIDA for docking with spike glycoprotein (PDB ID: 6vsb). (**a**) Standard ligand docked inside the receptor (HB in green color), (**b**) ligand inside the inner grid for validation, (**c**) Azithromycin docked peripherally, (**d**) Nitazoxanide docked with formation of weak HB (green color), and (**e**) Niclosamide docked with formation of strong HB (green color).

Docking of targeted drugs towards spike glycoprotein (PDB ID: 6vsb [18]) displayed the strength of Nitazoxanide, Niclosamide, and Azithromycin (Table 3). The co-crystalized ligand showed multiple HBs as peptidomimetic drugs, especially amino acids: ASN:422A (two HBs), GLN:493A, SER:494A, and TyR:495A (Figure 4a,b) (as reported). Azithromycin was detected with formation of HB with LYS:417A, however, it adopted a position far from the key peptide GLN:493A-TyR:495A (Figure 4c). Nitazoxanide binds strongly with formation of HB with ASN:422A through the NH amidic of aminothiazole (Figure 4d), while Niclosamide promoted HB interaction toward the critical peptide moiety (as displayed from standard ligand) with GLN:493A through its phenolic OH group (Figure 4e). This different binding mode of HB formation will affect drugs’ metabolism and subsequently their activity against SARS-CoV-2.

#### 2.4.2. Ligand Efficiency (LE) and Ligand lipophilic Efficiency (LLE) Scores

During drug repositioning, a drug undergoes the complete scenario of the drug development process. The assessment of ADMET (Absorption, Distribution, Metabolism, Excretion, and Toxicity) properties, especially the lipophilicity factor, is important in drug discovery and development. The affinity between the ligand and the target is a dominant parameter in drug discovery. Currently, validation of the molecular size, lipophilicity (cLogP), together with drugs’ activities (pIC_50_) using various helpful parameters designated as “optimization measures”, is required.

Ligand efficiency (LE) is used to determine the competence of drugs through calculation of its binding affinity (in terms of binding energy or pIC_50_) in relation to the number of heavy atoms in a molecule (number of non-hydrogen atoms (NHA)). LE analyses have practical utility in “lead optimization” towards a “drug-like candidate”, especially in a drug repurposing approach [19,20].

This tactic compares the affinity of drugs corrected for their size instead of considering the effectiveness or binding affinity of the whole structure. It is calculated as demonstrated in the following equations:

LE = ΔG ÷ NHA or LE = (pIC_50_ × 1.37) ÷ NHA

where ΔG = Gibb’s free energy, IC_50_ = half-maximal inhibitory concentration (in terms of molar concentration), and NHA = non-hydrogen atom. The recommended LE value should be in the range of 0.3. The preferred LE value should be higher than 0.3. The LE values for selected drugs are represented in Table 4.

Ligand Lipophilic Efficiency (LLE) offers a way to determine the affinity of a drug with respect to its lipophilicity. LLE is defined as the difference between the potency and cLogP, as explained in the following equation:

LLE = pIC_50_ − cLogP

The challenge in drug discovery is to improve the activity while keeping lipophilicity constant. For this, LLE is considered an effective and practical tool of keeping lipophilicity under control to avoid any “molecular obesity” during the drug optimization process. An acceptable lead drug should have LLE value ≥ 3, while LLE value ≥ 5 is recommended for drug-like candidates.

As shown in Table 4, Azithromycin displayed good LLE value followed by Niclosamide. On the other hand, and among the mentioned drugs, Azithromycin showed the lowest LE. Although Doxycycline had low potency, it had the highest LLE value, suggesting that this drug requires further optimization studies.

Celecoxib has the lowest LLE value. Piroxicam represented lipophilicity indices scores better than Celecoxib. These results suggest prescribing Piroxicam in combination with antibiotic in COVID-19 patients rather than Celecoxib. Regarding the rule of five values (Table 4), Azithromycin violates the rule of five as it has Mwt more than 500, and number of HBD and HBA more than five. Niclosamide, Nitazoxanide, Celecoxib, and Piroxicam obey the rule of five. Doxycycline showed violation in numbers of HBD and HBA. Azithromycin is eliminated in liver [21], it displayed low LE and violated the rule of five. These results indicate that patients with advanced stage of COVID-19 and compromised liver function may face problems with administration of Azithromycin [22,23].

### 2.5. Azithromycin Can Selectively Inhibit the Replication of SARS-CoV-2 Virus but Not MERS-CoV

**Figure 5:**
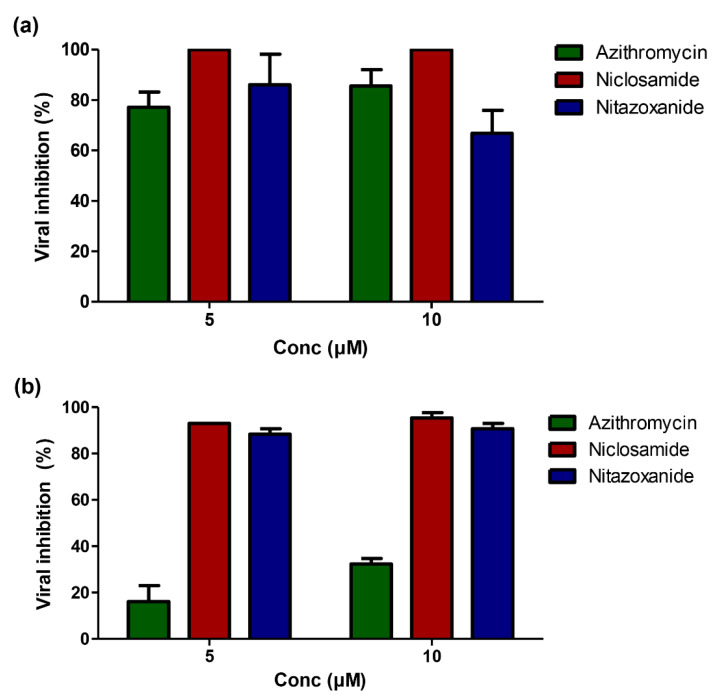
Differential anti-SARS-CoV-2 and Anti-MERS-CoV activities for Azithromycin, Niclosamide, and Nitazoxanide. (**a**) Anti-SARS-CoV-2 activity for Azithromycin, Niclosamide, and Nitazoxanide, as measured by Plaque reduction assay. (**b**) Anti-MERS-CoV activity for Azithromycin, Niclosamide, and Nitazoxanide, as measured by Plaque reduction assay.

To investigate whether the antiviral effect of the three FDA-approved drugs is common for other highly pathogenic coronaviruses or only selective against SARS-CoV-2, both SARS-CoV-2 and MERS-CoV viruses were treated with equal concentrations of the three drugs. Interestingly, Azithromycin showed a promising inhibitory activity against SARS-CoV-2 (viral inhibition is approximately 80% to 90%), compared to MERS-CoV (viral inhibition is approximately 20% to 30%) at the lowest (5 µM) and highest (10 µM) concentrations tested, respectively. However, Niclosamide and Nitazoxanide are equally effective against SARS-CoV-2 and MERS-CoV (Figure 5a,b).

### 2.6. Docking Study with MERS-CoV Viral Proteins

#### 2.6.1. Docking with the Main Protease

In order to examine the activity of these drugs against MERS-CoV virus, especially that Azithromycin displayed weak activity against MERS-CoV (Figure 5b), the docking protocol was employed here against the main protease of MERS-CoV (PDB ID: 4ylu [24]). Binding interaction was arranged as follow: Niclosamide with consensus score value 2, then Nitazoxanide with consensus score value 4, and finally, Azithromycin with consensus score value 6.

**Figure 6:**
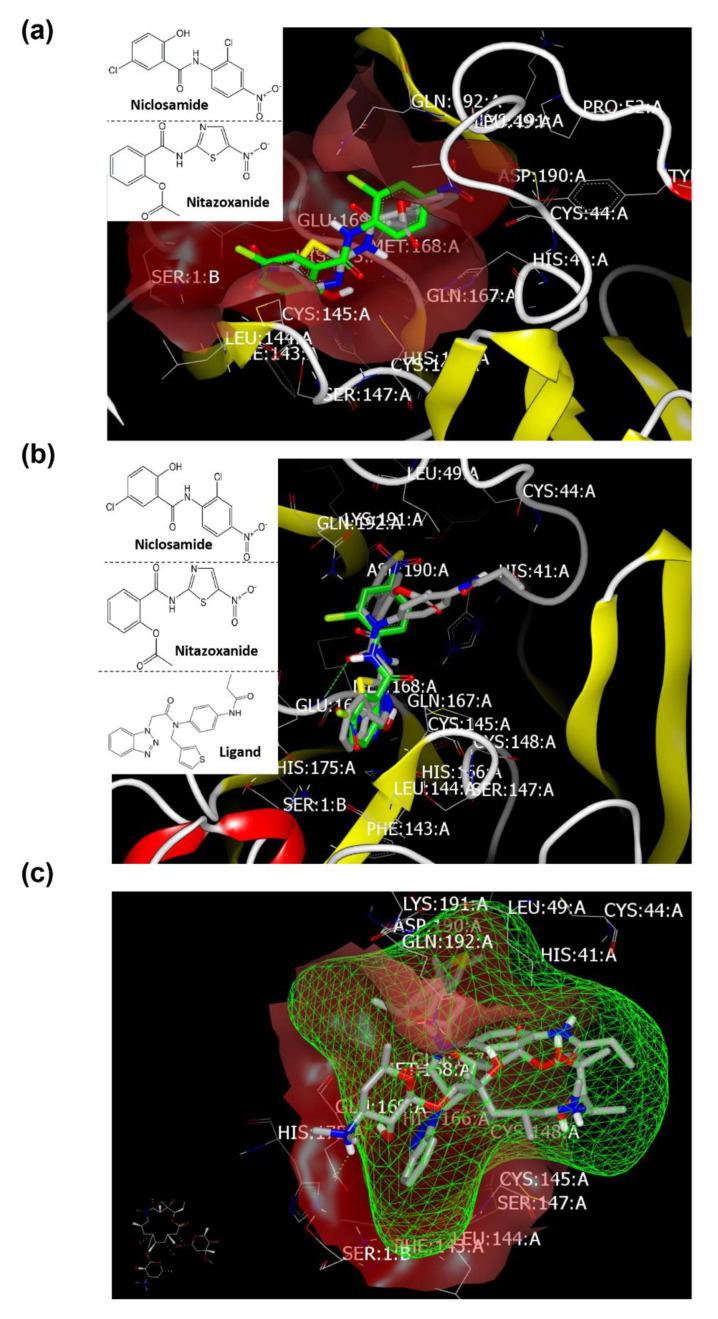
Visual representation by VIDA for docking with the main protease of MERS-CoV (PDB ID: 4ylu). (**a**) Niclosamide (green color) and Nitazoxanide (grey color) overlay each other, (**b**) ligand (grey color), Niclosamide (green color), and Nitazoxanide (grey color with yellow sulfur atom color) inside the inner grid for validation, and (**c**) Azithromycin with amino outside the grid.

Regarding their binding mode and pose, both Niclosamide and Nitazoxanide represented overlay to each other inside the active site, with capability of Nitazoxanide to form HB with GLN:167A (Figure 6a). Both exhibited high similarity inside the receptor compared to M^pro^ of SARS-CoV-2 (Figure 3b). Additionally, they exhibited a high degree of pose similarity with standard co-crystalized ligand. The ligand participated in HB with the NH peptide of GLU:169A (Figure 6b). Azithromycin occupied the receptor with formation of HB with the carboxylic functionality of GLU:169A. In comparison to the co-crystalized ligand, the amino group of Azithromycin bared outside the inner grid of the receptor (Figure 6c).

Interestingly, Azithromycin was completely buried inside the inner grid of the main protease of SARS-CoV-2 (Supplementary Figure S5). As a result, the volume of drugs and volume of the receptor of the main protease for SARS-CoV-2 and MERS-CoV could participate in directing the potency.

#### 2.6.2. Docking with the Spike Protein (PDB ID: 5x4r)

**Figure 7:**
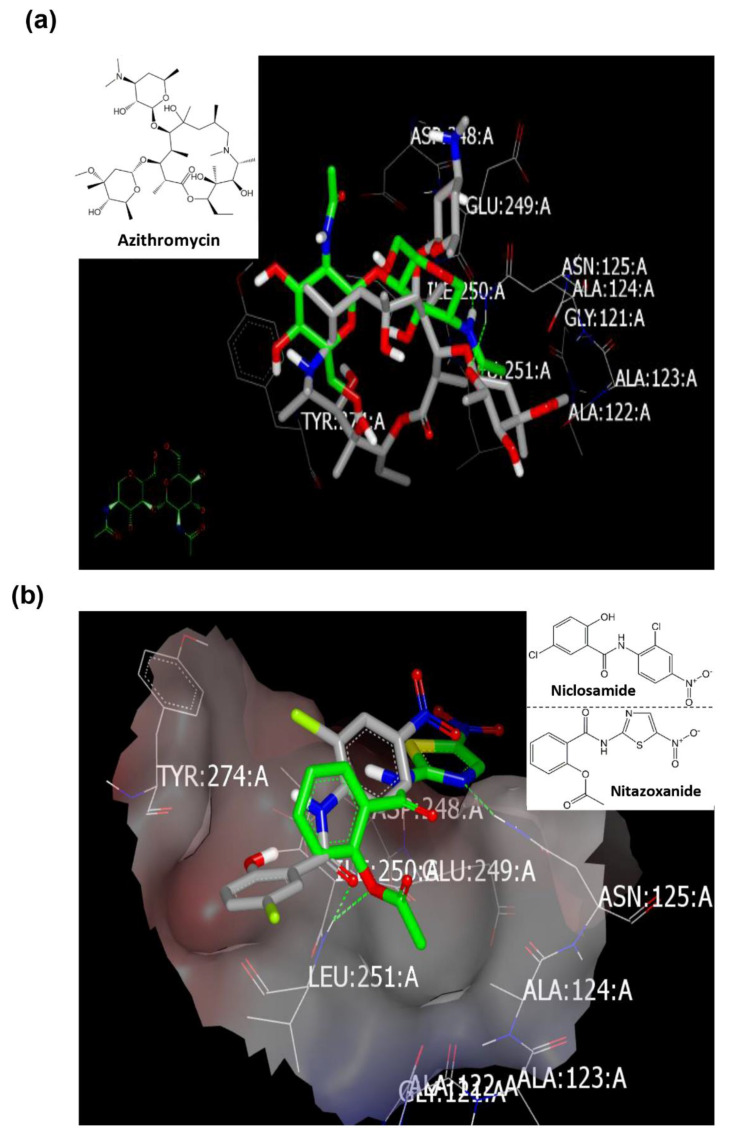
Visual representation by VIDA for docking with the main protease of MERS-CoV (PDB ID: 5x4r). (**a**) Azithromycin forms HB with ASN:125A, and (**b**) Niclosamide (grey color) and Nitazoxanide (green color) overlay each other.

The standard co-crystalized ligand deposited in the receptor with formation of HB with GLU:249A [25] (Supplementary Figure S6). Drugs showed binding strength order as follows: Nitazoxanide, Niclosamide, and Azithromycin with consensus scores 0, 5, and 7, respectively. Both Niclosamide (HB with LEU:251A) and Nitazoxanide (two HBs with ASN:125A and LEU:251A) overlay each other with the ligand (Figure 7b). Azithromycin occupied the receptor with formation of HB with ASN:125A (Figure 7b).

## 3. Discussion

Drug repositioning represents a promising approach to recognize off-label indications for formerly approved drugs that are different from their conventional medical uses. This approach offers the advantage of minimizing the required time, cost, and efforts for drug discovery process and safety evaluation. Furthermore, it reduces the risk of the drugs to fail, particularly due to safety issues since the majority of drugs are repositioned after verifying their safety in preclinical and clinical studies [9,10]. At the same time, drug repositioning demands an extensive study concerning the drug profile and new targeted disease mechanisms.

COVID-19 is considered a critical threat to the public health, and what aggravated the situation is that there is no existing antiviral therapy that is clinically approved for the management of this disease. Hence, the drug repositioning approach can be utilized as an opportunity for rapid screening of potential therapeutic options against SARS-CoV-2.

The current study signified numerous novel outcomes to be used as guidance or recommendations during the implementation of the FDA-approved drugs in the treatment protocol. Our results revealed that among the investigated drugs, Niclosamide, Azithromycin, and Nitazoxanide depicted the most potent antiviral activities against SARS-CoV-2 in Vero-E6 cells with IC_50_ values of 0.16, 0.32, and 1.29 µM, respectively. Regarding the mechanism of antiviral effect, the three examined drugs exhibited dual viral inhibition mechanisms. Both Azithromycin and Nitazoxanide exert their effects by virucidal action, while the antiviral effect of Niclosamide is mainly exerted through the inhibition of viral replication.

Additionally, the results of the in silico studies demonstrated strong binding affinity of the drugs to the viral main protease receptor in a descending order: Niclosamide, Nitazoxanide, and Azithromycin. On the other hand, the binding affinity of the tested drugs to the viral spike glycoprotein was in this descending order: Nitazoxanide, then Niclosamide, and Azithromycin. Furthermore, our results revealed that amongst all investigated drugs, Celecoxib showed the least LLE, thus the replacement of Celecoxib by Piroxicam in the treatment of COVID-19 patients is strongly suggested. Moreover, the administration of Azithromycin in advanced cases may cause problems to the patients as the drug violates the rule of five that is used to evaluate the drug-likeness. It is also noteworthy to mention that Azithromycin showed a selective inhibitory effect on SARS-CoV-2 but not on MERS-CoV, while Nitazoxanide and Niclosamide exhibited equal effects on both types of coronaviruses. These results are in accordance with the reported data in the literature that signified the broad antiviral activity spectrum of Nitazoxanide against various viruses [9]. In a recent study, Nitazoxanide has been reported to have in vitro antiviral activity against SARS-CoV-2 with IC_50_ of 2.12 μM [26], which is in accordance with our findings but with higher value (1.29 µM). Hence, researchers have suggested that this drug may be beneficial as a therapy for COVID-19 [27] considering not only the antiviral effect of Nitazoxanide but also its favorable inhibitory action against pro-inflammatory cytokines as well as its bronchodilatory effect [9]. Similarly, Niclosamide was reported to have antiviral inhibitory effects against a wide range of viruses such as Japanese encephalitis virus, MERS-CoV, Zika virus, hepatitis C virus, Ebola virus, Chikungunya virus, and human rhinoviruses [28]. Azithromycin was reported to have antiviral activities against many viruses such as SARS-CoV-2 [29], Zika virus [30], and Ebola virus [31]; however, the reported IC_50_ of Azithromycin against SARS-CoV-2 was 2.12 µM, which is higher than our results (0.32 µM) [29]. Herein, we propose Azithromycin, Niclosamide, and Nitazoxanide as novel and potent anti-SARS-CoV-2 drugs with potential therapeutic benefits in the treatment of COVID-19 patients. Besides, we provided preliminary data about FDA-approved drugs that could contribute in a dual mechanism to an anti-inflammatory response in patients with COVID-19 and partially hinder virus replication. We could show that Aspirin with anti-inflammatory, analgesic, antipyretic, and antithrombotic effects demonstrates antiviral activity against SARS-CoV-2. This finding is consistent with previous reports demonstrating that Aspirin has similar antiviral activity against different respiratory viruses such as human influenza viruses, rhinoviruses [32], human CoV-229E, and MERS-CoV in vitro [33]. The antiviral activity of Aspirin is probably cell-mediated by inhibiting prostaglandin (PG) and thromboxane synthesis via irreversible inactivation of both cyclo-oxygenase-1 (COX-1) and cyclo-oxygenase-2 (COX-2). Additionally, Aspirin modulates the nuclear factor kappa-light-chain-enhancer of activated B cells (NF-κB) pathway, downregulates the expression and activity of the inducible nitric oxide synthase (iNOS), inhibits oxidative phosphorylation uncoupling, and increases permeability of the mitochondrial membrane [34]. Similarly, Piroxicam is a potent, nonsteroidal, antipyretic, and anti-inflammatory agent that showed antiviral activity against NRC-03-nhCoV. Piroxicam has also shown antiviral activity against Herpes Simplex Virus type 1 (HSV-1) in vitro via direct interaction of Piroxicam with the viral particle before adsorption [35].

Recently, Chlorpheniramine maleate, a competitive histamine H1 receptor antagonist, showed potent antiviral activity against a broad spectrum of influenza viruses with IC_50_ of 3.56 and 11.84 μM. Accordingly, we showed that Chlorpheniramine maleate affected NRC-03-nhCoV at IC_50_ value of 3.6 µM. More recently, Westover and his colleagues reported a strong virucidal effect against SARS-CoV-2 of a nasal spray containing Chlorpheniramine maleate [36].

In conclusion, this study highlighted two commonly prescribed categories of FDA-approved drugs with specific members of potent antiviral activities against SARS-CoV-2. Those drugs, listed in this study, with potent antiviral activity, still need investigations in clinical trials to determine their actual in vivo activity in the treatment of COVID-19. Therefore, self-medication of COVID-19 patients with these drugs without clinical studies may be a high-risk practice and is not recommended.

## 4. Materials and Methods

### 4.1. Virus, Cells and FDA-Approved Drugs

Vero-E6 cells were maintained in Dulbecco’s Modified Eagle’s medium (DMEM) containing 10% Fetal Bovine Serum (FBS) (Invitrogen) and 1% Penicillin/Streptomycin (pen/strep) antibiotic mixture at 37 °C, 5% CO_2_. To generate virus stock, cells were distributed into tissue culture flasks 24 h prior to infection with hCoV-19/Egypt/NRC-3/2020 isolate at a multiplicity of infection (MOI) of 0.1 in infection medium (DMEM containing 2% FBS, 1% pen/strep, and 1% L-1-tosylamido-2-phenylethyl chloromethyl ketone (TPCK)-treated trypsin. Two hours later, the infection medium containing virus inoculum was removed and replaced with fresh infection medium and incubated for three days. At the indicated time point, cell supernatant was collected and centrifuged for 5 min at 2500 rpm to remove small particulate cell debris. The supernatant was transferred to fresh 50 mL falcon tube, aliquoted, and titrated using the plaque infectivity assay.

The tested FDA-approved drugs, listed in Table 1 and Table 2, were kindly granted by the Egyptian International Pharmaceutical Industries “EIPICO”, the Holding Company for Pharmaceuticals, Chemicals, and Medical Appliances “HoldiPharma”, and the National Organization for Drug Control and Research in Egypt.

### 4.2. MTT Cytotoxicity Assay

To assess the half maximal cytotoxic concentration (CC_50_), stock solutions of the test compounds were prepared in 10% DMSO in ddH_2_O and diluted further to the working solutions with DMEM. The cytotoxic activity of the extracts was tested in Vero-E6 cells by using the 3-(4, 5-dimethylthiazol -2-yl)-2, 5-diphenyltetrazolium bromide (MTT) method with minor modifications. Briefly, the cells were seeded in 96-well plates (100 µL/well at a density of 3 × 105 cells/mL) and incubated for 24 h at 37 °C in 5% CO_2_. After 24 h, cells were treated with various concentrations of the tested compounds in triplicates. After 24 h, the supernatant was discarded, and cell monolayers were washed with sterile 1× phosphate buffer saline (PBS) 3 times, and MTT solution (20 µL of 5 mg/mL stock solution) was added to each well and incubated at 37 °C for 4 h followed by medium aspiration. In each well, the formed formazan crystals were dissolved with 200 µL of acidified isopropanol (0.04 M HCl in absolute isopropanol = 0.073 mL HCL in 50 mL isopropanol). Absorbance of formazan solutions was measured at λmax 540 nm with 620 nm as a reference wavelength using a multi-well plate reader. The percentage of cytotoxicity compared to the untreated cells was determined with the following equation.

The plot of % cytotoxicity versus sample concentration was used to calculate the concentration which exhibited 50% cytotoxicity (TC_50_) [37]:

$$\%\;\mathrm{cytotoxicity}=\frac{\left(\mathrm{absorbance}\;\mathrm{of}\;\mathrm{cells}\;\mathrm{without}\;\mathrm{treatment}-\mathrm{absorbance}\;\mathrm{of}\;\mathrm{cells}\;\mathrm{with}\;\mathrm{treatment}\right)\;\times \;100}{\mathrm{absorbance}\;\mathrm{of}\;\mathrm{cells}\;\mathrm{without}\;\mathrm{treatment}\;}$$

### 4.3. Plaque Infectivity Assay

For the titration of hCoV-19/Egypt/NRC-03/2020 (NRC-03-nhCoV) (Accession Number on GSAID: EPI_ISL_430820), the plaque infectivity assay was carried out as previously described [38] with minor modifications. Briefly, the propagated virus was serially diluted 10-folds in medium without FBS. A volume of 100 µL of each individual virus dilution was mixed with 400 µL of infection medium and used to inoculate 80–90% confluent Vero-E6 cell monolayers. Control well was included in the same plate and was inoculated with 500 µL of serum-free medium. The plate was then incubated at 37 °C under 5% CO_2_ for 1 h to allow virus adsorption and rocked every 15 min to ensure homogenous exposure of the cells to infection and avoid drying of cells. After 1 h, the virus inoculum was discarded and the cell monolayers were overlaid with 3 mL of DMEM plus 0.6% agarose containing 1 μg/mL of TPCK-treated trypsin, 10% FBS, and 1× pen/strep, and the appropriate concentration of the test drug. To allow the solidification of the agarose component of the overlayer medium, the plate was left at room temperature (RT) for 10 min then incubated at 37 °C under 5% CO_2_. After 72 h, 1 mL of fixation solution (10% formalin) was added to each well for 1 h for cell fixation and virus inactivation. The fixer was later discarded, and the plate wells were flushed with water and dried. For visualization of the plaques, 1 mL of the staining solution (0.1% crystal violet) was added to each well for 5 min, dye was discarded, and the plate wells were rinsed in water and dried. Viral plaques appeared as clear unstained spots (due to viral infection) in a violet (stained cells) background. The virus titer was calculated through the following equation:

Plaque forming unit (PFU) per mL=Number of plaques×inoculated volume of the virus×virus dilution×10

### 4.4. Plaque Reduction Assay

To assess the preliminary antiviral activity of the studied FDA-approved drugs, the plaque reduction assay [39] was carried out in a six-well plate, where Vero-E6 cells (1.2 × 10^6^ cells) were cultivated for 24 h at 37 °C. The NRC-03-nhCoV virus was diluted to give 102 plaque forming units (PFU)/well and mixed with the safe concentration of the tested compounds and incubated for 1 h at 37 °C before being added to the cells. Growth medium was removed from the cell culture plates and the cells were inoculated with (100 µL/well) virus with the tested compounds. After 1 h of virus adsorption, 3 mL of DMEM supplemented with the overlay medium with the indicated concentrations of the tested compounds were added onto the cell monolayers. The plates were left to solidify and incubated at 37 °C until formation of viral plaques for 3 days. Cell fixing solution was added for 1 h, then plates were stained with 0.1% crystal violet in distilled water. Control wells were included, where untreated virus was incubated with Vero-E6 cells, and finally, plaques were counted and percentage reduction in plaques formation in comparison to control wells was recorded as following:

(1) $$\mathrm{Percent}\;\mathrm{of}\;\mathrm{reduction}=\frac{\mathrm{untreated}\;\mathrm{virus}\;\mathrm{count}\;-\;\mathrm{treated}\;\mathrm{virus}\;\mathrm{count}\;}{\mathrm{untreated}\;\mathrm{viral}\;\mathrm{count}}\times \;100$$

### 4.5. Inhibitory Concentration 50 (IC_50_) Determination

In 96-well tissue culture plates, 2.4 × 10^4^ Vero-E6 cells were distributed in each well and incubated overnight in a humidified 37 °C incubator under 5% CO_2_ condition. The cell monolayers were then washed once with 1× PBS and subjected to virus adsorption for 1 h at room temperature (RT). The cell monolayers were further overlaid with 50 μL of DMEM containing varying concentrations of the selected test compounds, Azithromycin, Niclosamide, and Nitazoxanide. Following incubation at 37 °C in 5% CO_2_ incubator for 72 h, the cells were fixed with 100 μL of 4% paraformaldehyde for 20 min and stained with 0.1% crystal violet in distilled water for 15 min at RT. The crystal violet dye was then dissolved using 100 μL absolute methanol per well and the optical density of the color measured at 570 nm using Anthos Zenyth 200 rt plate reader (Anthos Labtec Instruments, Heerhugowaard, Netherlands). The IC_50_ of the compound is that required to reduce the virus-induced cytopathic effect (CPE) by 50%, relative to the virus control.

### 4.6. In Vitro Inhibition of Replication Efficiency at Different Virus Concentrations

Confluent Vero-E6 cells’ monolayers were infected with NRC-03-nhCoV in triplicate at MOI of 0.005 and 0.001 at 37 °C. The inocula were removed at 1 h post-infection (hpi), cell monolayers were washed with 1× PBS, and overlaid with infection media (1× DMEM supplemented with 1% Pen/Strep, 0.3% bovine serum albumin (BSA), and 2 µg/mL TPCK-treated trypsin). The cell culture supernatants were collected at 48 hpi. The virus titer was determined with plaque infectivity assay.

### 4.7. Mechanism of Action(s)

To investigate whether the tested drugs (Azithromycin, Niclosamide, and Nitazoxanide) with high selectivity index and potent activity against NRC-03-nhCoV affect (a) viral adsorption, (b) viral replication, or (c) viricidal effect, the plaque infectivity reduction assay was performed according to the following protocols.

#### 4.7.1. Viral Adsorption Mechanism

The viral adsorption mechanism was assayed according to a protocol by Zhang and his colleagues [40] with minor modifications. Vero-E6 cells were cultivated in a 6-well plate (105 cells/mL) for 24 h at 37 °C. Each tested drug was applied in 200 µL medium without supplements and co-incubated with the cells for 2 h at 4 °C. The inocula containing the non-absorbed drug were removed by washing cells three successive times with supplement-free medium. SARS-CoV-2 virus diluted to 104 PFU/well was co-incubated with the pretreated cells for 1 h, and then 3 mL DMEM supplemented with 2% agarose were added. Plates were left to solidify and then incubated at 37 °C to allow the formation of viral plaques. The plaques were fixed and stained as described above to calculate the percentage reduction in plaque formation compared to control wells, which comprised untreated Vero-E6 cells directly infected with NRC-03-nhCoV.

#### 4.7.2. Viral Replication Mechanism

The impact of tested drug on viral replication was determined as previously described [41]. Vero-E6 cells were cultivated in a 6-well plate (10^5^ cell/mL) for 24 h at 37 °C. Virus was inoculated directly to the cells and incubated for 1 h at 37 °C. The inocula containing the non-adsorbed viral particles were removed by washing cells three successive times with supplement-free medium. The test compound was added in varying concentrations to infected cells for another 1 h contact time. After removing the inocula containing the tested drug, 3 mL of DMEM supplemented with 2% agarose were added to the cell monolayer. Plates were left to solidify and incubated at 37 °C until the appearance of viral plaques. Cell monolayers were fixed in 10% formalin solution for 1 h and stained with crystal violet. Control wells contained Vero-E6 cells incubated with the virus. Plaques were counted and the percentage reduction in plaque formation was compared to the control wells.

#### 4.7.3. Virucidal Mechanism

The virucidal mechanism was assayed following a previously described protocol [42]. In a 6-well plate, Vero-E6 cells were cultivated (10^5^ cells/mL) for 24 h at 37 °C and 200 µL of serum-free DMEM containing SARS-CoV-2 was added to each sample with promising inhibition. After 1 h incubation, the mixture was diluted 10-fold three times using serum-free medium, which still allowed viral particles to grow on Vero-E6 cells. Next, 100 µL of each dilution were added to the Vero-E6 cell monolayer. After 1 h contact time, a DMEM overlayer was added to the cell monolayer. Plates were left to solidify and incubated at 37 °C to allow the formation of viral plaques. The plaques were fixed and stained as described above to calculate the percentage reduction in plaque formation. This value was compared to control wells comprising cells infected with virus and not pretreated with the tested material.

### 4.8. In Silico Analyses

#### 4.8.1. Molecular Modeling

The X-ray crystal structure coordinates of SARS-CoV-2 main protease (M^pro^) were retrieved from PDB (PDB ID: 6yef [17] and 6lu7 [43]) in addition to the retrieved receptor for S glycoprotein (PDB ID: 6vsb [18]) with their co-crystallized bound ligand α-ketoamide, N3, and ligand 1, respectively (Figure 2). The docking study was performed using OpenEye scientific software version 2.2.5 (SantaFe, NM, USA, http://www.eyesopen.com). For the validation of the docking study, the co-crystal-bound ligands were redocked. Both structures exhibited high similarity and overlaid each other, as reported in our previous work [16].

#### 4.8.2. Physiochemical Parameter and Lipophilicity Calculations

Drugs’ parameters including cLogP were calculated according to their practical values as reported in CHEMBL, Drug Bank, and PubChem free access websites. Lipinski’s rule (Rule of five) was calculated by the free access website https://www.molsoft.com/servers.html.

### 4.9. Statistical Analyses

All experiments were performed in three biological repeats. Statistical tests and graphical data presentation were carried out using GraphPad Prism 5.01 software. Data are presented as the average of the means. The IC_50_ and CC_50_ curves represent the nonlinear fit of “Normalize” of “Transform” of the obtained data, their values were calculated using GraphPad prism as “best fit value”.

## Figures and Tables

**Table 1:** Antiviral activity of anti-microbial and anti-inflammatory FDA-approved drugs against NRC-03-nhCoV.

(A) Anti-microbial FDA-approved drugs				
FDA-approved drug	Initial indication	CC_50_ (Vero-E6)	IC_50_ (NRC-03-nhCoV)	SI
**Amikacin sulphate**	**Aminoglycoside antibiotic**	**2456 µM**	**16.81 µM**	**146.10**
**Azithromycin**	**Macrolide-type antibiotic**	**793 µM**	**0.32 µM**	**2478.13**
Amoxicillin	Penicillin-type antibiotic	614.57 µM	16.12 µM	38.12
Benzathine penicillin	Long-acting penicillin antibiotic	728.2 µM	15.78 µM	46.15
Chloramphenicol	Broad-spectrum bacteriostatic	33.92 µM	16.94 µM	2.00
Cefotaxime	Third-generation cephalosporin antibiotic	3155 µM	42.72 µM	73.85
Cephalexin	First-generation cephalosporin antibiotic	522.95 µM	13.17 µM	39.71
Ceftriaxone	Third-generation cephalosporin antibiotic	445.91 µM	16.14 µM	27.63
Cefoperazone	Third-generation cephalosporin antibiotic	69.03 µM	12.36 µM	5.58
**Ceftazidime**	**Third-generation cephalosporin antibiotic**	**5554 µM**	**46.14 µM**	**120.37**
Clindamycin	Lincosamide antibiotic	436.45 µM	15.67 µM	27.85
Ciprofloxacin	Fluoroquinolone antibiotic	3516 µM	61.62 µM	57.06
**Doxycycline**	**Tetracycline antibiotic**	**636.1 µM**	**5.1 µM**	**124.73**
Flucloxacillin	Narrow-spectrum penicillin-type antibiotic	966.23 µM	157.78 µM	6.12
**Levofloxacin**	**Fluoroquinolone antibiotic**	**2156 µM**	**13.84 µM**	**155.78**
Linezolid	Narrow-spectrum oxazolidinone antibiotic	816.5 µM	16.3 µM	50.1
**Moxifloxacin**	**Fluoroquinolone antibiotic**	**2242 µM**	**12.23 µM**	**183.32**
Nitrofurantoin	Narrow-spectrum antibiotic	599.113 µM	16.22 µM	36.94
Neomycin	Aminoglycoside antibiotic	833.1 µM	18.12 µM	45.98
**Niclosamide**	**Anthelminthic and antibacterial drug**	**204.61 µM**	**0.16 µM**	**1278.81**
**Nitazoxanide**	**Broad-spectrum anti-infective drug**	**665.15 µM**	**1.29 µM**	**515.62**
Nystatin	Antifungal medication	182.64 µM	160.85 µM	1.14
(B) Analgesics and antipyretics				
FDA-approved drug	Initial indication	CC_50_ (Vero-E6)	IC_50_ (NRC-03-nhCoV)	SI
**Acetyl Salicylic acid “Aspirin”**	**Anti-inflammatory and antipyretic**	**1255 µM**	**12.16 µM**	**103.21**
Paracetamol	Analgesic and antipyretic	4980 µM	≥IC_50_	<1
Celecoxib	Nonsteroidal anti-inflammatory drug (NSAID)	140.37 µM	13.02 µM	10.78
Ciclesonide	Glucocorticoid used to treat asthma and rhinitis	119.5 µM	4.2 µM	28.73
**Chlorpheniramine maleate**	**Antihistamine used to treat allergic rhinitis**	**465.65 µM**	**3.6 µM**	**129.35**
Dexamethasone	Anti-inflammatory corticosteroid medication	1901 µM	122.55 µM	15.51
Diclofenac sodium	Nonsteroidal anti-inflammatory drug (NSAID)	138.31 µM	96.24 µM	1.44
Fluticasone Propionate	Synthetic glucocorticoid to treat asthma and COPD	32.04 µM	1.71 µM	18.74
Formoterol Fumarate	Long-acting bronchodilator	568.63 µM	71.8 µM	7.92
Hydrocortisone	Anti-inflammatory glucocorticoid	614 µM	7.1 µM	87.10
Indomethacin	Nonsteroidal anti-inflammatory drug	671.7 µM	8.51 µM	78.93
Ibuprofen	Nonsteroidal anti-inflammatory drug	1166 µM	88.71 µM	13.14
Ketoprofen	Nonsteroidal anti-inflammatory drug	822.62 µM	21.5 µM	38.31
Ketorolac Tromethamine	Nonsteroidal anti-inflammatory drug	2042 µM	153.42 µM	13.31
Metamizole sodium	Analgesic and antipyretic	947.5 µM	14.97 µM	63.29
Montelukast	Leukotriene receptor antagonist to treat asthma	9.86 µM	2.7 µM	3.65
Meloxicam	Nonsteroidal anti-inflammatory drug	262.16 µM	12.4 µM	21.21
Methylprednisolone	Glucocorticoid anti-inflammatory medication	3344 µM	90.44 µM	36.97
Naphazoline	Decongestant	636.1 µM	9.52 µM	66.82
**Piroxicam**	**Nonsteroidal anti-inflammatory drug**	**1795 µM**	**8.21 µM**	**218.64**
Salmeterol	Long-acting bronchodilator	4.1 µM	1.5 µM	2.73

Abbreviations: “CC_50_” half maximal cytotoxic concentration; “IC_50_” half maximal inhibitory concentration; “SI” Safety index; “COPD” Chronic obstructive pulmonary disease. Bold: FDA-approved drugs with high selectivity index (SI ≥ 100).

**Table 2:** Mechanisms of action of FDA-approved anti-microbial drugs with promising antiviral activity for repurposing against COVID-19.

Name of Compound	Conc. (µM)	Mode of Action *		
		Viral Adsorption	Viral Replication	Virucidal
Azithromycin	1.3	31%	4%	51%
	0.64	27%	2%	51%
	0.322	2%	0%	34%
	0.16	0%	0%	12%
Niclosamide	10.4	0%	70%	37%
	5.2	0%	68%	21%
	2.6	0%	55%	21%
	1.302	0%	23%	16%
Nitazoxanide	10.4	11%	Toxic	78%
	5.2	11%	Toxic	75%
	2.6	1%	40%	61%
	1.302	0%	35%	39%

* The mechanism of action of the three compounds were done at concentrations higher than the half-maximal inhibitory effect “IC_50_” to better resolve the mechanism of action.

**Table 3:** Binding mode of most active drugs with their consensus score against spike glycoprotein and M^pro^.

Name of Compound	Spike Glycoprotein		Main Protease			
	6vsb	Binding Interaction	6y2f	Binding Interaction	6lu7	Binding Interaction
Azithromycin	173	HB with LYS:417A	153	No HB formations	197	HBs with GLU:166A and GLN:189A. Fully occupied receptor domains with two terminal HBs formation.
Niclosamide	153	HB with GLN:493A	153	HB with GLN:192A	113	No HB formation. The phenolic moiety oriented deeply in the pocket domain.
Nitazoxanide	150	HB with ASN:422A	96	HB with MET:165A	134	No HB formation. The salicyloyl moiety oriented deeply in the pocket domain.

Abbreviations: HB (Hydrogen Bond); LYS (Lysine); GLU (Glutamic acid); GLN (Glutamine); ASN (Asparagine); MET (Methionine); A (Alanine).

**Table 4:** Summary of ligand efficiency scores of the target drugs against SARS-CoV-2.

	Rule of Five (RO5)				cLogP	Experimental Data			
	Mwt	NHA	HBA	HBD		IC_50_ (µM)	pIC_50_	LE	LLE
Azithromycin	749.00	52	14	5	2.44	0.32	6.49	0.17	4.05
Niclosamide	327.12	21	4	2	2.95	0.16	6.79	0.44	3.84
Nitazoxanide	307.28	21	7	1	2.12	1.29	5.89	0.38	3.77
Celecoxib	381.38	26	4	1	4.01	13.02	4.89	0.26	0.88
Piroxicam	331.35	23	5	2	3.1	8.21	5.09	0.30	1.99
Doxycycline	444.44	32	9	6	−0.7	5.1	5.29	0.22	5.99

NHA: non-hydrogen atom = heavy atom; HBA: hydrogen bond acceptor; HBD: hydrogen bond donor; RO5: rule of thumb to evaluate drug likeness or determine if a chemical compound with a certain pharmacological or biological activity has chemical properties and physical properties that would make it likely orally active; LE: Ligand efficiency; LLE: Ligand Lipophilic Efficiency; IC_50_: half maximal inhibitory concentration; pIC_50_: Negative log of the IC_50_ value.

## Supplementary Materials

The following are available online at https://www.mdpi.com/1424-8247/13/12/443/s1, Figure S1: Half maximal cytotoxic concentration “CC_50_” of the tested anti-microbial drugs in Vero-E6 cells. Various dilutions of the drugs were applied to the 90% confluent cell monolayers and assayed after 72 h with MTT assay. The half maximal cytotoxic concentrations “CC_50_” were calculated using nonlinear regression analysis of GraphPad Prism software (version 5.01) by plotting log inhibitor versus normalized response (variable slope). Figure S2: Dose-response curves for anti-inflammatory, antipyretic, and anti-asthmatic FDA-approved drugs as determined by MTT assay. The half maximal cytotoxic concentrations “CC_50_” were calculated using nonlinear regression analysis of GraphPad Prism software (version 5.01) by plotting log inhibitor versus normalized response (variable slope). Figure S3: Half maximal inhibitory concentration “IC_50_” of the tested anti-microbial drugs against NRC-03-nhCoV virus in Vero-E6 cells. Various dilutions of the drugs were applied to the 90% confluent cell monolayers and assayed after 72 h with crystal violet assay. The half maximal inhibitory concentrations “IC_50_” were calculated using nonlinear regression analysis of GraphPad Prism software (version 5.01) by plotting log inhibitor versus normalized response (variable slope). Figure S4: Half maximal inhibitory concentration “IC_50_” for anti-inflammatory, antipyretic, and anti-asthmatic FDA-approved drugs against NRC-03-nhCoV virus in Vero-E6 cells. Various dilutions of the drugs were applied to the 90% confluent cell monolayers and assayed after 72 h with crystal violet assay. The half maximal inhibitory concentrations “IC_50_” were calculated using nonlinear regression analysis of GraphPad Prism software (version 5.01) by plotting log inhibitor versus normalized response (variable slope). Figure S5: Azithromycin inside the grid receptor of M^pro^ of SARS-CoV-2. Figure S6: Co-crystalized ligand of MERS-CoV Spike protein ID: 5x4r” (a) Within the inner grid, and (b) Inside the receptor.
