# Supplementary figures and images for: FDA-Approved Drugs with Potent In Vitro Antiviral Activity against Severe Acute Respiratory Syndrome Coronavirus 2
Source: Pharmaceuticals (Basel). 2020 Dec 4;13(12):443. doi: 10.3390/ph13120443 (PMC7761982; doi:10.3390/ph13120443)

Supplementary Figure (1S)

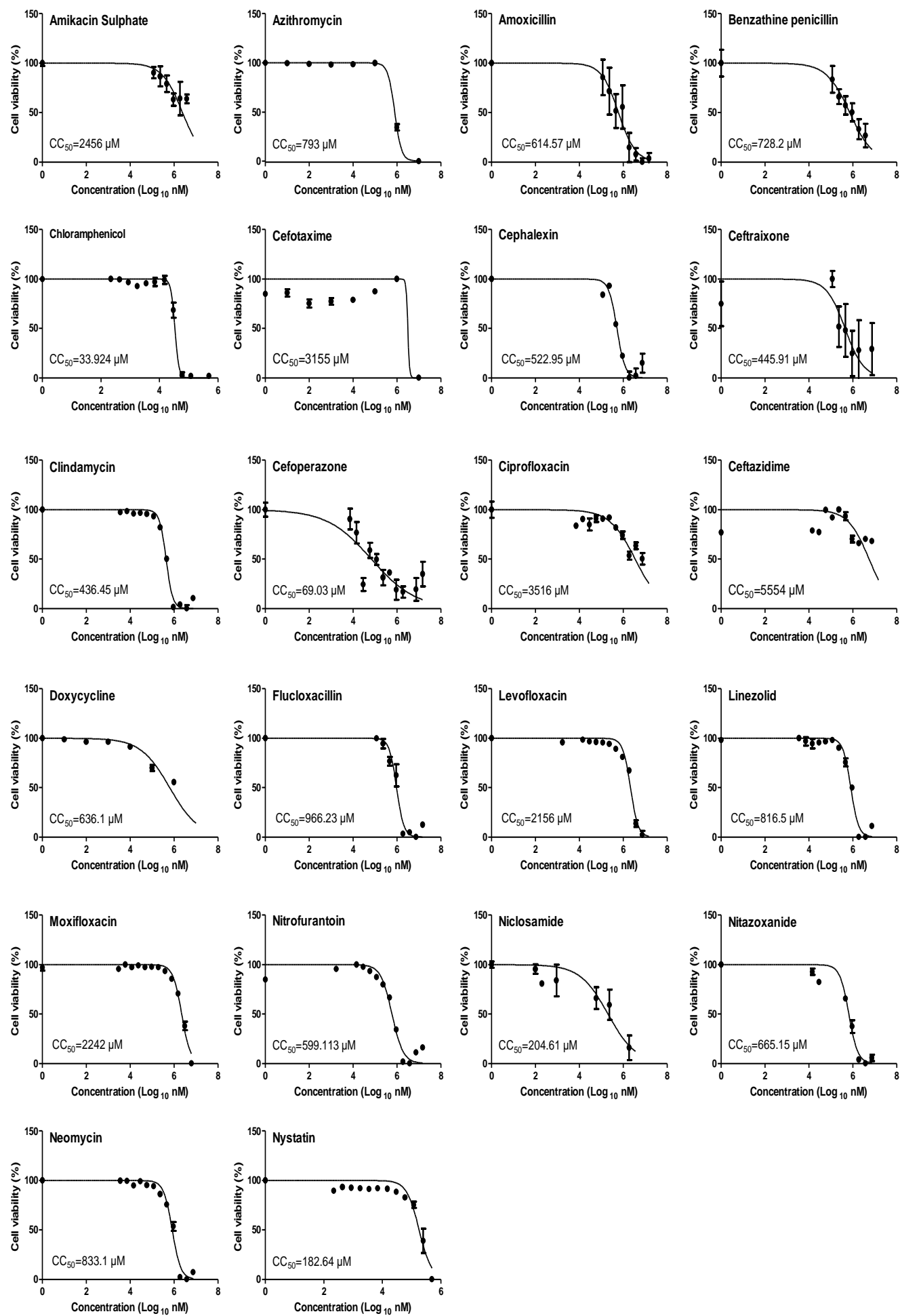

Supplement: Supplementary file 1 [file pharmaceuticals-13-00443-s001.zip › Supplementary Figure S1-R.pdf]

Supplementary figure (2S)

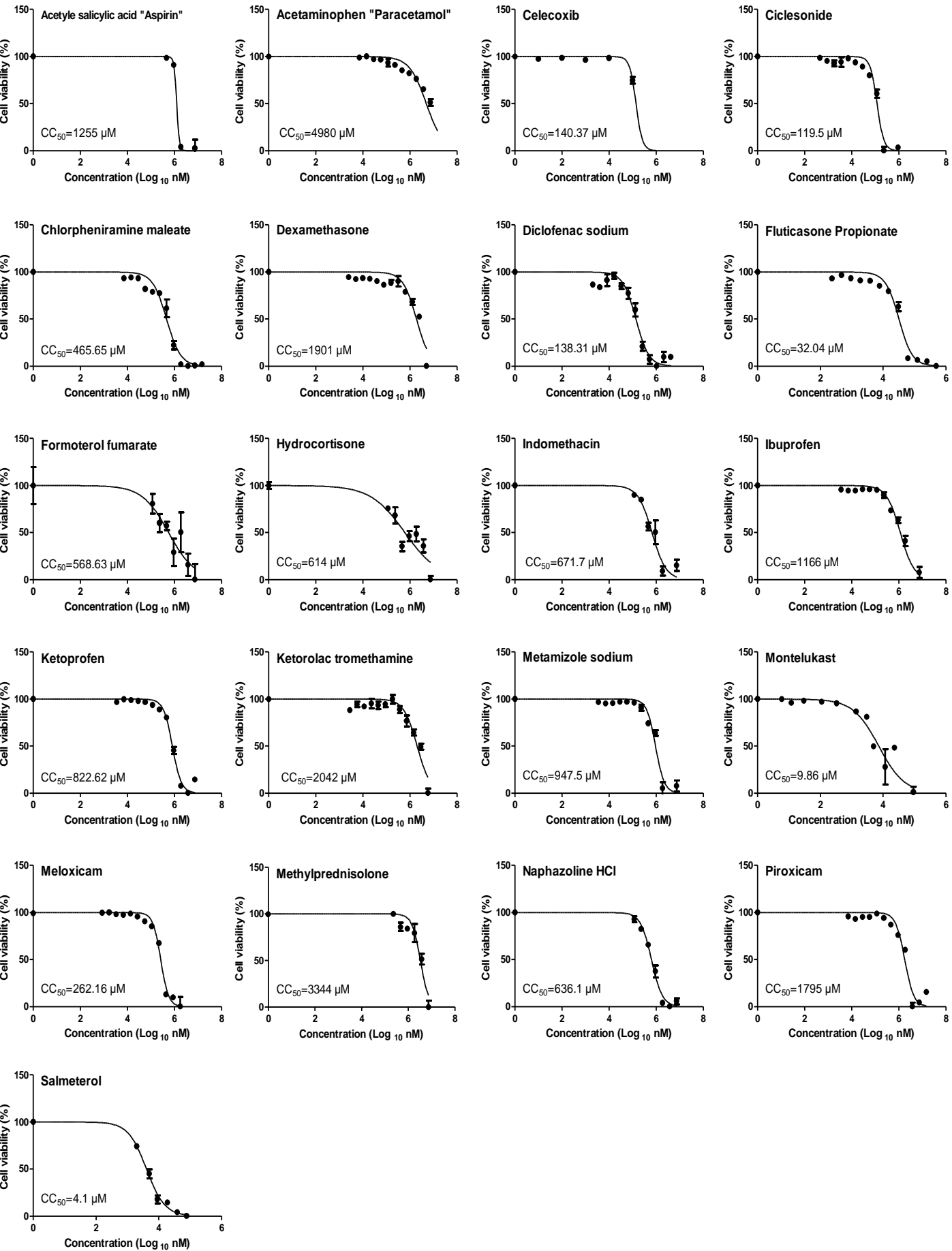

Supplement: Supplementary file 1 [file pharmaceuticals-13-00443-s001.zip › Supplementary Figure S2-R.pdf]

Supplementary figure (3S)

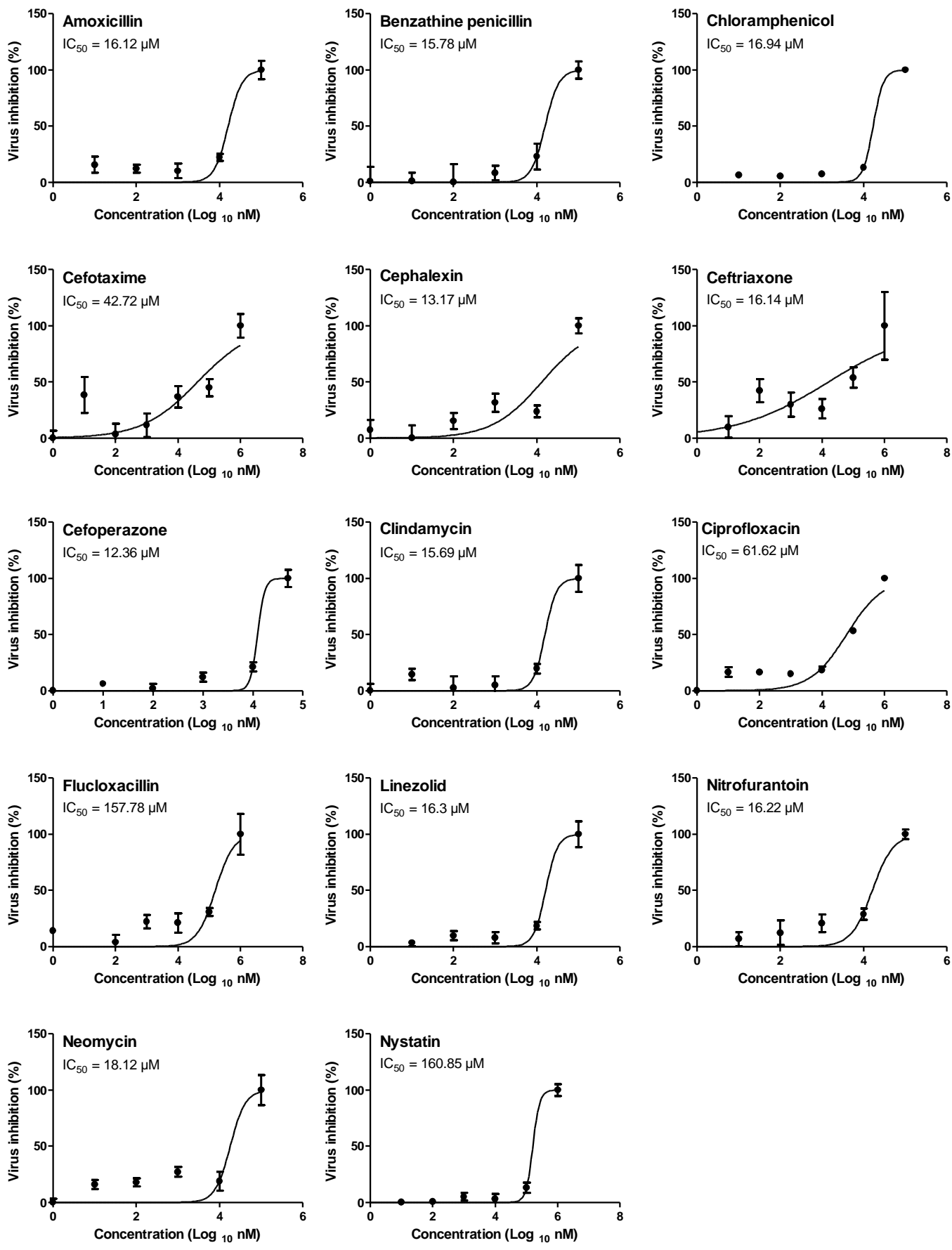

Supplement: Supplementary file 1 [file pharmaceuticals-13-00443-s001.zip › Supplementary Figure S3-R.pdf]

Supplementary figure (4S)

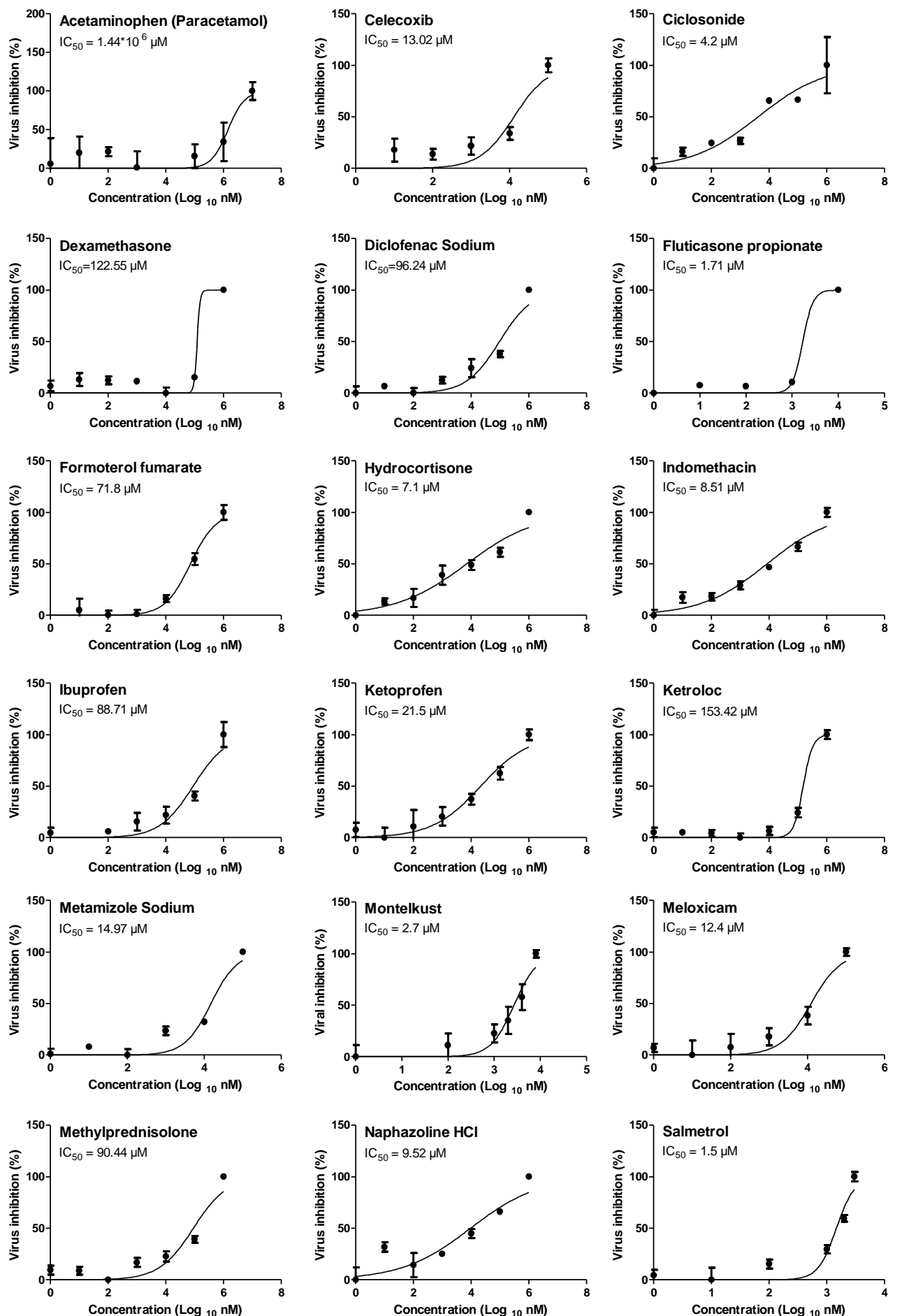

Supplement: Supplementary file 1 [file pharmaceuticals-13-00443-s001.zip › Supplementary Figure S4-R.pdf]
